# Microdermabrasion facilitates direct current stimulation by lowering skin resistance

**DOI:** 10.1002/ski2.76

**Published:** 2021-11-15

**Authors:** P. Y. Chhatbar, S. Liu, V. Ramakrishnan, M. S. George, S. A. Kautz, W. Feng

**Affiliations:** ^1^ Department of Neurology Duke University School of Medicine Durham North Carolina USA; ^2^ Department of Neurology Tiantan Hospital Capital Medical University Beijing China; ^3^ Department of Public Health Sciences Medical University of South Carolina Charleston South Carolina USA; ^4^ Psychiatry and Behavioral Science Brain Stimulation Laboratory College of Medicine Charleston South Carolina USA; ^5^ Department of Health Science & Research College of Health Professions Medical University of South Carolina Charleston South Carolina USA; ^6^ Ralph H. Johnson VA Medical Center Charleston South Carolina USA

## Abstract

**Background:**

Transcranial direct current stimulation (tDCS) is reported to induce irritating skin sensations and occasional skin injuries, which limits the applied tDCS dose. Additionally, tDCS hardware safety profile prevents high current delivery when skin resistance is high.

**Objective:**

To test if decreasing skin resistance can enable high‐dose tDCS delivery without increasing tDCS‐related skin sensations or device hardware limits.

**Methods:**

We compared the effect of microdermabrasion and sonication on 2 mA direct current stimulation (DCS) through forearm skin for 2–3 min on 20 subjects. We also surveyed the subjects using a questionnaire throughout the procedure. We used a linear mixed‐effects model for repeated‐measures and multiple logistic regression, with adjustments for age, race, gender and visit.

**Results:**

Microdermabrasion, with/out sonication, led to significant decrease in skin resistance (1.6 ± 0.1 kΩ or ∼32% decrease, *p* < 0.0001). The decrease with sonication alone (0.4 ± 0.1 kΩ or ∼7% decrease, *p* = 0.0016) was comparable to that of sham (0.3 ± 0.1 kΩ or ∼5% decrease, *p* = 0.0414). There was no increase in the skin–electrode interface temperature. The perceived DCS‐related sensations did not differ across skin preparation procedures (*p* > 0.16), but microdermabrasion (when not combined with sonication) led to increased perceived sensation (*p* < 0.01).

**Conclusions:**

Microdermabrasion (with/out sonication) resulted in reduced skin resistance without increase in perceived skin sensations with DCS. Higher current can be delivered with microdermabrasion‐pre‐treated skin without changing the device hardware while reducing, otherwise higher voltage required to deliver the same amount of current.

## Graphical abstract

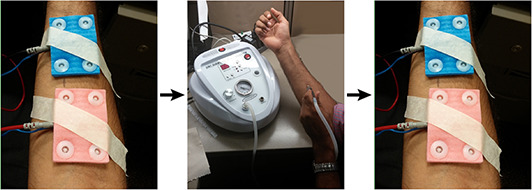

1

**What's already known about this topic?**

- Direct current stimulation (DCS), when applied transcranially, has promised in a variety of neuropsychiatric conditions but underdosing might be the reason for variability in efficacy across research subjects and groups
- Applying higher currents is demonstrated to be safe for brain in animal studies, but can lead to uncomfortable skin sensations in humans and may increase the risk of skin injury

**What does this study add?**

- Skin preparation procedure by microdermabrasion, with or without sonication, leads to decrease in skin resistance by about 32%
- Decreased skin resistance results in increase of about 50% injected current at the same applied voltage
- Microdermabrasion does not lead to significant changes in sensations related to DCS

**What is the translational message?**

- Microdermabrasion leads to more efficient delivery of DCS by facilitating higher amount of current at the same applied voltage without changes in skin sensations related to DCS

**What are the clinical implications of this work?**

- As more research groups are pushing for higher amperage of DCS for a variety of neuropsychiatric disorders, microdermabrasion can help injecting higher currents without increasing applied voltage and without increasing discomfort to patients related to such stimulation

## 2 INTRODUCTION

Electrical stimulation methods, such as transcranial direct current stimulation (tDCS), have been investigated in a variety of neuropsychiatric conditions including depression, post‐surgery pain control, stroke recovery, etc. With the current human tDCS protocols, safety profiles are good, although skin injuries have been reported. However, efficacy is not yet consistent, and there is significant inter‐individual variability in tDCS response.^1^, ^2^, ^3^ One possible reason for this inconsistent response to tDCS in certain subjects is lower ‘delivered dose’ to the brain. In a published meta‐analysis and meta‐regression, we have demonstrated a positive dose–response relationship between tDCS‐related parameters and reduction in post‐stroke upper extremity motor impairment.^4^ The data also suggested that this dose–response relationship may extend beyond the present dose range. Therefore, higher dose maybe needed, but safety can be a concern. Human studies have shown that tDCS delivery up to 4 mA is safe in stroke^5^ and healthy subjects.^6^

In order to deliver a constant current, required voltage is different across individuals depending on the individual's skin resistance. Electrically less conductive skin can prevent delivery of higher direct current with potentially unsafe voltage gradient across the electrodes. Stratum corneum—the outermost layer of the skin—consists of compressed and desiccated skin cells, which contributes to a significant fraction of electrical resistance to the body. While the rest of the body typically has resistance of ∼500 Ω, skin has resistance in the range of ∼10–100 kΩ.^7^, ^8^ Overall, high skin resistance is a hurdle to deliver a high current and the drop in resistance can be attributed to electroporation of appendageal ducts at low voltage (<30 V) and also lipid‐corneocyte matrix as voltage gradient is further raised (>30 V) across the skin.^9^ While voltage is not an issue at current <4 mA doses for current tDCS devices, higher doses at ten(s) of milliamps might pose hardware limitations on tDCS devices.

We hypothesize that partial removal of stratum corneum either by physical abrasion^10^ and/or sonication^11^ may decrease the electrical resistance of the skin. This skin pre‐treatment can improve the penetration of direct current and decreases the need for higher voltage gradient across the DCS electrodes. In this study, we aimed to compare the effect of microdermabrasion, sonication, combination of both, with sham skin preparation procedure on skin resistance and perceived sensations to DCS. We used forearm skin, instead of scalp, for DCS because a recent report suggested that sensation levels on arm were similar to those reported on the head.^12^

## 3 MATERIALS AND METHODS

### 3.1 Study subjects

The protocol was approved by the local Institutional Review Board, and all the procedures were performed at Medical University of South Carolina. All subjects signed the informed consent before participating in the study. We included adult subjects (18 years or older) of any gender or race who are able and willing to provide an informed consent. The exclusion criteria were (1) any unhealed cuts or wounds on forearm skin; (2) exposed skin of any forearm with diseases including, but not limited to psoriasis, eczema, allergies, insect bites, blisters, etc.; (3) history of keloid formation; and (4) presence of any DCS risk factors, for example, an electrically, magnetically or mechanically activated metal or non‐metal implants including cardiac pacemaker or any other electrically sensitive support system; non‐fixed metal in forearm or arm; pregnancy (unknown effects of DCS on foetus); pre‐existing skin lesion, bone defect or forearm condition.

### 3.2 Study design

We investigated the effect of microdermabrasion (yes/no) and sonication (yes/no) on DCS delivery in this study. Therefore, four sessions were required to test all four permutations of these procedures (microdermabrasion, sonication, both, none/sham). We achieved this by having participants make two visits, with two sessions at each visit (one session on each forearm), totalling four skin preparation procedures (Table 1). The assignment code of each procedure for a given forearm at a given visit was generated using a computer‐generated pseudo‐random block‐design algorithm (MATLAB; Mathworks). Four unique patterns of procedure assignment were generated so that each pattern was allocated to five subjects. We administered DCS via an iontophoresis device (Chattanooga Ionto^TM^ Dual Channel Iontophoresis System, Chattanooga Group) before and after skin preparation procedure. This ensured that each subject serves as their own control for the given procedure, in that the DCS application before the skin preparation procedure served as a baseline DCS. We monitored the applied voltage, injected current, and temperature at anode and cathode using a data acquisition device (DI‐245, Dataq Instruments). We performed skin preparation procedures using a generic diamond microdermabrasion machine that includes ultrasound therapy setup (Kendal HB‐MF02). We surveyed the subjects at various stages of the session using questionnaire and also examination of forearm at the site of DCS application.

**TABLE 1:** Enrolment and study plan

Activity	Consent day	Visit 1 (can be same as consent day)		Visit 2 (at least 7 days apart from visit 1)	
		Left forearm	Right forearm	Left forearm	Right forearm
Consent and random allocation of four skin preparation procedures (30 min)^a^	x				
Pre‐procedure DCS		x	x	x	x
Skin preparation procedure^b^		x	x	x	x
Post‐procedure DCS		x	x	x	x

^a^ Consent can be obtained on the same day as Visit 1.

^b^ four skin preparation procedures are allocated through a pseudorandom block design—see Table 2 for assignment.

### 3.3 Study procedures

At each visit, we inspected the subject's each forearm to ensure that no exclusion criteria were met. We asked the subjects to wash forearms with soap and water to remove any ointment, lotion, oil or debris. We applied saline‐soaked pads (0.9% NaCl w/v) with DCS electrodes (5 × 7 cm^2^ sponge pads, Soterix Medical) on the palmer aspect of one forearm with anode on proximal site, cathode on distal site, and distance of at least 2 cm between the two, making sure that the circuit is not ‘shorted’ through the wet skin between the electrodes. We inserted a T‐type thermocouple (TC‐TWB2‐12, Dataq Instruments) at the skin–electrode interface to monitor temperature.

1. *Pre‐procedure DCS*: We applied 2 mA of DC current for about 3 min (totalling 5 mA·min of charge) where current was ramped up and ramped down over 30 s at the beginning and at the end of the stimulation, respectively. Ramping of the current can minimize the skin sensation or discomfort associated with current injection. We sampled applied voltage gradient, injected current and skin–pad interface temperature at 50 samples/s throughout the DCS application. We calculated resistance (*R*) from the applied voltage (*V*) and injected current (*I*) using Ohm's law: *R* = *V* ÷ *I*.
2. *Skin preparation procedure:* At the end of DC stimulation, we removed the pads after marking the pad locations and dried the skin with a paper towel or soft towelettes. We prepared the skin with one of the following four options as assigned for a given visit at a given forearm:
  ‐ *Microdermabrasion* (*procedure A*): We gently rubbed a diamond microdermabrasion probe on the skin area where the electrode was positioned using a slow, linear repetitive motions at 5 cm/s speed and at 30 cmHg suction, repeated thrice. The procedure took about 1 min.
  ‐ *Sonication* (*procedure B*): We applied a flat ultrasound probe of 45 mm diameter with a sonically conductive gel or lotion and moved over the skin area with slow, linear repetitive motions at 5 cm/s speed with 1 W/cm^2^ power and 3 MHz frequency for 5 min.
  ‐ *Both* (*procedure C*): We started the procedure with microdermabrasion and followed it by sonication as described above.
  ‐ *Sham* (*procedure D*): We did not perform any skin preparation. We waited about 5 min to match the procedure time in A or B or C.

As mentioned earlier, at the end of each procedure above, we cleaned the skin with a paper towel or soft towelettes.

3. *Post‐procedure DCS:* We administered DCS again for 3 min per the procedure outlined above.

#### 3.3.1 Subjective sensations data collection and processing

Throughout the protocol, we surveyed the subjects for subjective sensations like itching, tingling, burning, electric shock‐like sensation and also monitored skin redness (through visual observation by the study procedure administrator). We further quantified the severity of perceived sensations (or observation of skin redness) as none (0), mild (1), moderate (2) or severe (3). The time points of the survey were before, during and after pre‐skin preparation DCS; during skin preparation procedure; before, during and after post‐skin preparation DCS (Table S1). Since the majority of the subjects have mild level of tingling or itching, we reduced the dimensionality of the data by summing all the perceived sensations (itching, burning, etc.) with their severity (mild, moderate, etc.) and came up with an aggregate number for perceived subjective sensations at each time point for each skin preparation procedure administered on the individual subjects.

#### 3.3.2 Biometric data collection and processing

Resistance and temperature data were first imported from native DAQ file format into raw values using MATLAB (Mathworks). Values at the end of DCS were used for statistical analysis. Using values at the end of DCS ensured that the temperature and resistance have reached to stable values and therefore minimized the variability that one might encounter during the early time points of DCS.

#### 3.3.3 Data analysis

We used the value of body resistance and skin temperature at the end of DCS to minimize the routinely observed variability in these values at the beginning of the stimulation. We used the difference in the values that are collected during each DCS session applied before and after each skin preparation procedure. We analysed data with aggregate subjective sensations using SAS V9.4 (SAS Inc), applying multiple logistic regression for categorical outcomes and linear mixed‐effects models for continuous repeated measures. We present least squares means and contrasts from these analyses, for interpretations of the results.

## 4 RESULTS

Twenty healthy adult subjects were consented, enrolled and randomized to one of the four unique sequences of skin preparation procedures (Table 2). All of them completed the study protocol without anyone dropping out of the study.

**TABLE 2:** Subject characteristics and randomization assignments

Subject ID	Age	Gender	Race	Hispanic	Randomization^a^
1	28	M	Asian	N	BCDA
2	26	F	Asian	N	DABC
3	35	M	Asian	N	ADCB
4	27	F	Black	N	BCDA
5	30	M	White	N	BCDA
6	28	F	White	N	BCDA
7	29	M	Asian	N	ADCB
8	43	M	White	Y	ADCB
9	32	F	White	N	DABC
10	35	M	White	N	CBAD
11	41	M	Asian	N	CBAD
12	29	F	White	N	CBAD
13	23	F	White	N	CBAD
14	24	F	Asian	N	DABC
15	26	M	Asian	N	CBAD
16	39	M	White	N	DABC
17	22	F	White	N	ADCB
18	26	F	White	Y	BCDA
19	35	F	White	N	ADCB
20	27	M	Asian	N	DABC

^a^ Randomization column presents four letters, each representing a unique procedure (A = Microdermabrasion; B = Sonication; C = Both; D = Sham). First through four letter shows procedure offered at left forearm during the first visit, right forearm during the first visit, left forearm during the second visit, right forearm during the second visit, respectively.

### 4.1 Microdermabrasion, but not sonication, led to a significant decrease in skin resistance (Figure 1, Table 3)

Microdermabrasion itself or with sonication led to an average of 1.6 ± 0.1 kΩ (adjusted *p* < 0.0001) reduction in skin resistance. Sonication, on the other hand, led to only slight change the skin resistance (−0.4 ± 0.1 kΩ, adjusted *p* = 0.002). Sham procedure also slightly changed the skin resistance (−0.3 ± 0.1 kΩ, adjusted *p* = 0.041). In the linear mixed‐effect model, race (*p* = 0.73), age (*p* = 0.81), gender (*p* = 0.17) or visit time (*p* = 0.33) were not associated with the changes in skin resistance.

**FIGURE 1:**
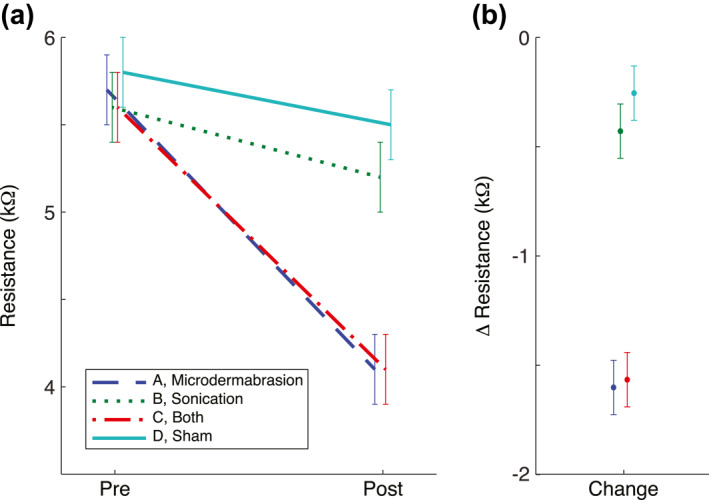
Microdermabrasion, but not sonication, leads to a decrease in skin resistance. Microdermabrasion with (*p* < 0.0001) or without (*p* < 0.0001) sonication significantly decreased the skin resistance. (a) Pre = End of Pre‐procedure DCS; Post = end of post‐procedure DCS. (b) Change = difference between Pre and Post. Error bars: SEM. Values are adjusted means

**TABLE 3:** Comparison of change in body resistance between the skin preparation procedures presented as differences of least square means (LSM)

Pr1	Pr2	Estimate	Standard error	DF	*t*‐Value	Pr>|*t*|	Alpha	Lower	Upper
D	B	0.1740	0.1683	19	1.03	0.3141	0.05	−0.1782	0.5262
D	A	1.3464	0.1683	19	8.00	**<0.0001**	0.05	0.9942	1.6985
D	C	1.3109	0.1683	19	7.79	**<0.0001**	0.05	0.9587	1.6630
B	A	1.1724	0.1683	19	6.97	**<0.0001**	0.05	0.8202	1.5245
B	C	1.1369	0.1683	19	6.76	<0.0001	0.05	0.7847	1.4891
A	C	−0.03550	0.1683	19	−0.21	0.8352	0.05	−0.3877	0.3167

*Note*: Pr1 and Pr2 are the skin preparation procedures the subjects have received (A = Microdermabrasion; B = Sonication; C = Both; D = Sham). Bold lettering signify *P* values less than 0.05 (alpha) or, in other words, 95% probability of the findings not observed by chance.

### 4.2 None of the skin preparation procedures caused significant rise in skin temperature

Skin temperature did not increase after skin preparation procedures. The linear mixed‐effects model, which adjusted gender, race, age and visit time, procedure type (at anode, *p* = 0.75; at cathode, *p* = 0.87) or interactions between procedure types and time points did not find significance (at anode, *p* = 0.22; at cathode, *p* = 0.45).

### 4.3 Subjects experienced reversible skin redness but no skin injuries

No subjects had any visible injury or breach in the skin barrier as a result of DCS and skin preparation procedures. We did observe transient skin redness on both forearms after DCS in all of the patients, which disappeared within minutes to hours. Specific to the skin preparation procedures, we observed that all 20 subjects had skin redness after microdermabrasion, 7 subjects after sonication, 16 subjects after both procedures and 8 subjects after none (sham).

### 4.4 Subjects did not have significantly different perception of sensations during DCS but microdermabrasion procedure itself can induce increased sensations

Many subjects reported sensations like tingling, itching, burning, etc., both during DCS procedures and skin preparation procedures. The perceived sensations were not different across skin preparation procedures or sham during the DCS after the skin preparation procedure when compared to the DCS before the skin preparation procedure (*p* > 0.15, Table 4). Perceived sensations were higher after microdermabrasion procedure when compared with sonication or sham (*p* < 0.01, Table 5).

**TABLE 4:** Comparison of change in perceived sensations during DCS before/after the skin preparation procedures presented as differences of least square means (LSM)

Pr1	Pr2	Estimate	Standard error	DF	*t*‐Value	Pr > |*t*|	Alpha	Lower	Upper
D	B	−0.2000	0.2406	19	−0.83	0.4162	0.05	−0.7036	0.3036
D	A	−0.05000	0.2406	19	−0.21	0.8376	0.05	−0.5536	0.4536
D	C	−0.3500	0.2406	19	−1.45	0.1621	0.05	−0.8536	0.1536
B	A	0.1500	0.2406	19	0.62	0.5404	0.05	−0.3536	0.6536
B	C	−0.1500	0.2406	19	−0.62	0.5404	0.05	−0.6536	0.3536
A	C	−0.3000	0.2406	19	−1.25	0.2276	0.05	−0.8036	0.2036

*Note*: Pr1 and Pr2 are the skin preparation procedures the subjects have received (A = Microdermabrasion; B = Sonication; C = Both; D = Sham).

**TABLE 5:** Comparison of perceived sensations during the skin preparation procedures themselves presented as differences of least square means (LSM)

Pr1	Pr2	Estimate	Standard error	DF	*t*‐Value	Pr > |t|	Alpha	Lower	Upper
D	B	0.05000	0.1377	19	0.36	0.7206	0.05	−0.2383	0.3383
D	A	−0.4000	0.1377	19	−2.90	**0.0091**	0.05	−0.6883	−0.1117
D	C	−0.1000	0.1377	19	−0.73	0.4766	0.05	−0.3883	0.1883
B	A	−0.4500	0.1377	19	−3.27	**0.0041**	0.05	−0.7383	−0.1617
B	C	−0.1500	0.1377	19	−1.09	0.2897	0.05	−0.4383	0.1383
A	C	0.3000	0.1377	19	2.18	0.0422	0.05	0.01173	0.5883

*Note*: Pr1 and Pr2 are the skin preparation procedures the subjects have received (A = Microdermabrasion; B = Sonication; C = Both; D = Sham). Bold lettering signify *P* values less than 0.05 (alpha) or, in other words, 95% probability of the findings not observed by chance.

## 5 DISCUSSION

We quantitatively demonstrated that microdermabrasion reduced skin resistance by 1.6 kΩ (∼32%) on average, which could allow almost 50% increase in current delivery at a given voltage gradient. We believe that partial removal of stratum corneum, as a result of microdermabrasion, leads to a decrease in skin resistance and is consistent with a previous report.^10^ Sonication, on the other hand, did not lead to reduction in skin resistance and was inconsistent to the previous report.^11^ It could be attributable to the lower power (1 W/cm^2^ vs. 12 W/0.8 cm^2^) and/or higher frequency (3 MHz vs. 55 kHz).^11^ Microdermabrasion, sonication or combination did not cause skin injury or any adverse event. None of these procedures increased skin temperature. There is no difference in perceived sensations among the four skin preparation groups.

This proof‐of‐concept study has important implications to future tDCS research. Lowering skin resistance can substantially decrease the need of higher voltage gradient, which can in turn reduce the odds of electrical stimulation‐related skin injuries. There is a high likelihood that future tDCS applications would use >5 mA currents which would require device to generate higher voltage gradients between anode and cathode in order to deliver requested tDCS currents. For example, an individual with skin resistance of 4 kΩ, a voltage of 32 V is required to deliver a constant direct current at 8 mA; however, only 24 V is required to deliver the same 8 mA current if the skin is pre‐treated with microdermabrasion. This may also improve the safety profile of tDCS if high dose is required for certain disease conditions.

We would like to emphasize that dermabrasion needs to be uniform and must evenly abrade the stratum corneum throughout the area of DCS application. Uneven dermabrasion, on the other hand, may lead to ‘selective shunting’ where current may pass through only the part of the skin and increase the probability of skin injury. The latter is the reason why it is not safe to apply tDCS on injured skin. We observed that more subjects had skin redness at anode during the second visit as compared to the first visit. An interval of 7 days between the visits may not be long enough to have epidermal turnover, which can take up to 6 weeks.^13^

This study is not free from limitations. First, we chose to use forearm skin, rather than scalp, mainly for the practicality of the study. Forearm skin has different physical characteristics when compared to the scalp. However, perceived sensations from DCS between arm and head were recently demonstrated to be comparable.^12^ Regardless, our data should be interpreted with caution when extrapolating to the tDCS study where currents are applied to the scalp. Second, reported sensations (mild, moderate and severe) are subjective measurements with limitations. The open‐label nature of the procedures can also influence the perceived sensations by the subjects, and it may increase the risk of bias. Therefore, quantification of the perceived sensations reported by different subjects should be considered with caution. Third, the younger age of subjects in our study (22–43 years) may not be representative of several disease conditions where DCS is intended as a therapeutic tool. For example, tDCS is a potential neuro‐modulatory therapy in stroke patients who are typically much older. Older subjects have shown decrease in sensitivity to electrical stimulation in another study.^14^ However, we note that the stimulation duration was 50 ms (vs. minutes in case of tDCS) and skin thinning from microdermabrasion might lead to higher sensations in older population when compared to younger population. With the limited number of subjects (*n* = 20), we could not do age adjustment while controlling for gender, race and ethnicity due to insufficient statistical power. Fourth, duration of DCS used in present study (∼3 min) is an order of magnitude shorter than used for therapeutic purposes (10–40 min). We chose this duration in the light of plateau effect with decrease of body resistance and in the interest of keeping the visit duration to 1 h. Given the encouraging results of microdermabrasion of this study, the next step is to do scalp microdermabrasion before tDCS in elderly population. We may also test the high current at 4 mA or more with a longer duration (20+ min).

## 6 CONCLUSION

This study quantitatively assessed the impact of three skin preparation procedures on the skin resistance. The use of microdermabrasion is a simple and effective method to reduce the skin resistance. Additionally, there is no significant difference in subjective sensations or discomforts to DCS as a result of this skin preparation. Our next logical step is to replicate this study on the scalp, which may have an important implication in future transcranial DCS research.

## CONFLICT OF INTEREST

The authors declare no conflict of interests.

## AUTHOR CONTRIBUTIONS

**P. Y. Chhatbar:** Conceptualization; Formal analysis; Writing – original draft; Writing – review & editing. **S. Liu:** Formal analysis; Writing – original draft; Writing – review & editing. **V. Ramakrishnan:** Data curation; Writing –original draft; Writing – review & editing. **M. S. George:** Writing – original draft; Writing – review & editing. **S. A. Kautz:** Writing – original draft; Writing – review & editing. **W. Feng:** Funding acquisition; Writing – original draft; Writing – review & editing.

## ETHICS STATEMENT

The protocol was approved by the local Institutional Review Board and all the procedures were performed at Medical University of South Carolina. All the subjects who participated in the study provided a written informed consent.

## Supporting information

TABLE S1

## Data Availability

The data that support the findings of this study are available from the corresponding author upon reasonable request.
