# Supplementary material for: Microdermabrasion facilitates direct current stimulation by lowering skin resistance
Source: Skin Health Dis. 2021 Nov 15;2(3):e76. doi: 10.1002/ski2.76 (PMC9435456; doi:10.1002/ski2.76)
Supplement: Supplementary file 1 — TABLE S1 [file SKI2-2-e76-s001.pdf]

Effect of Different Skin Preparations on Delivery of Direct Current Stimulation

Subject ID: ________________ **Study Assignment:** **Visit 1:** Left: _____ Right: _____
Visit 2: Left: _____ Right: _____

Anode (+, red): Palmar / Dorsal, Proximal / Distal **Cathode (-, black):** Palmar / Dorsal, Proximal / Distal

Fill or Rate the Following: (*Severity Scale*: **0**: absent; **1**: mild; **2**: moderate; **3**: severe)

Intervention	**Microdermabrasion (A)**							**Sonication (B)**						
Date														
Session	**Pre-Prep DCS**			**Prep**	**Post-Prep DCS**			**Pre-Prep DCS**			**Prep**	**Post-Prep DCS**		
Time														
Skin effects	Pre	Dur	Post	Dur	Pre	Dur	Post	Pre	Dur	Post	Dur	Pre	Dur	Post
Tingling														
Itching														
Burning														
Pain														
Electric Shock														
Skin Redness (**Anode**/+/Red)		N/A				N/A			N/A				N/A	
Skin Redness (**Cathode**/–/Black)		N/A				N/A			N/A				N/A	
Other														

Intervention	**Microdermabrasion+Sonication (C)**							**Sham (D)**						
Date														
Session	**Pre-Prep DCS**			**Prep**	**Post-Prep DCS**			**Pre-Prep DCS**			**Prep**	**Post-Prep DCS**		
Time														
Skin effects	Pre	Dur	Post	Dur	Pre	Dur	Post	Pre	Dur	Post	Dur	Pre	Dur	Post
Tingling														
Itching														
Burning														
Pain														
Electric Shock														
Skin Redness (**Anode**/+/Red)		N/A				N/A			N/A				N/A	
Skin Redness (**Cathode**/–/Black)		N/A				N/A			N/A				N/A	
Other														

Notes:
